# Interpretative Phenomenological Study Exploring Why People With Kidney Failure Say ‘No’ to a Kidney Transplant

**DOI:** 10.1111/jan.70301

**Published:** 2025-10-21

**Authors:** Emma Jones, Leah McLaughlin, Kate Shakespeare, Jane Noyes

**Affiliations:** ^1^ Cardiff and Vale University Health Board Cardiff UK; ^2^ Bangor University School of Health Sciences Bangor UK; ^3^ Cadwaladr University Health Board Renal Unit Glan Clwyd Hospital Rhyl UK

**Keywords:** decline, interpretive phenomenological inquiry, kidney disease, kidney transplant, nursing, patient experience, qualitative, shared decision‐making

## Abstract

**Aim:**

To develop an in‐depth understanding of peoples' perceptions and experiences of decision‐making and reasons why they declined the opportunity of a kidney transplant.

**Design:**

The Theory of Planned Behaviour informed the qualitative interpretative phenomenological analysis.

**Methods:**

Semi‐structured interviews were conducted between August 2022 and June 2023 with thirty adults in the United Kingdom who had declined a kidney transplant. Interviews were digitally recorded and transcribed verbatim.

**Findings:**

Deciding against having a kidney transplant for the majority of people was a concrete decision. Multiple reasons transcended four cross cutting themes: *The impact of negative past experiences on kidney transplant decision‐making, Negative attitudes, beliefs, and perceptions towards kidney transplantation, Preferred not to have a kidney transplant, and Perceived benefits of deciding against a kidney transplant.* Earlier negative experiences culminated in mistrust. People feared kidney transplant failure and were not willing to take the risk of being worse off. Some people perceived they were too old and preferred younger people to be offered available kidneys. COVID‐19 negatively impacted some people's decisions.

**Conclusion:**

Despite people's decisions being perceived as at odds with healthcare professionals and current policies to increase transplantation rates, overall, the decision not to have a kidney transplant appeared carefully thought through.

**Implications for the Profession and/or Patient Care:**

People's choices were informed, multifaceted and shaped by personal experiences, perceived risks and individual values. Recognising these factors is essential in improving patient‐centred care and shared decision‐making. Nurse‐led patient education needs to carefully balance promoting kidney transplant as the preferred kidney replacement treatment option.

**Impact:**

The findings contribute new understanding and theory as to why people living with kidney failure decide against having a kidney transplant. Perceived benefits of not having a transplant outweighed potential advantages, and patients exercised their legal right to make an informed decision.

**Reporting Method:**

COREQ and SRQR.

**Patient or Public Contribution:**

People living with kidney disease were involved from the outset; their contributions included prioritising the research question, shaping the study design, commenting on participant documents, analysing, interpreting findings and dissemination.

**Summary**

- What does this paper contribute to the wider global clinical community?
  ○ This study represents the first large‐scale and rigorous qualitative exploration of why people in the UK declined kidney transplantation despite being medically suitable candidates.
  ○ The study highlights the need for more patient‐centred decision‐support aids and the need for implementation of available shared decision‐making tools.
  ○ The findings and refined theory can be used by healthcare professionals to further develop patient assessments, interactions and education resources that include more accessible tailored information on risks and benefits of kidney transplantation, and as a continuous quality improvement of care initiative to counter patients' negative experiences that impact on their transplant decision‐making.

## 1 Introduction

Kidney transplantation is recognised as the most cost‐effective and clinically beneficial treatment for people living with kidney failure (Chaudhry et al. 2022; UK Renal Registry 2024). International policies further promote increased access to transplantation by standardising organ donation systems and expanding international living donation programmes to ensure equitable access to kidney transplantation (International Society of Nephrology 2024; The United States Department of Health and Human Services (US HSS) 2024). From a health economics perspective, kidney transplantation is significantly more cost‐effective than dialysis (Kidney Research UK 2023). However, little is known about why some individuals choose not to pursue transplantation despite being eligible. Studies exploring the views, experiences and decision‐making preferences of people living with kidney failure in relation to declining transplantation are limited, and findings are often reported briefly within broader research on kidney replacement therapies.

## 2 Background

In the United Kingdom (UK), policies prioritise increasing organ transplant uptake to improve patient survival and quality of life while reducing reliance on dialysis (NHS Blood and Transplant 2020). *The Organ Donation and Transplantation 2030: Meeting the Need* report outlines strategies to improve access to both living and deceased kidney transplantation, aligning with Welsh policy recommendations (NHS Wales 2022). Kidney patient education is usually delivered by specialist kidney nurses to support and help patients understand information to inform their kidney replacement treatment choice. Members of the kidney specialist interdisciplinary team (social workers, psychologists, doctors, pharmacists) work together providing information and specialist support to people.

### 2.1 Kidney Transplant Assessment and Access

Clinical guidelines recommend starting assessment for kidney transplantation approximately 1 year before kidney replacement treatment is required (British Transplant Society 2011; NICE Guidelines 2018). Successful placement on the kidney transplant waiting list involves extensive medical screening for comorbidities, malignancies and viral infections, as well as psychological and social evaluations. While individuals are expected to receive comprehensive information about their treatment options, it is often assumed that all eligible patients will choose kidney transplantation. It is unclear how many individuals decline kidney transplantation while they are otherwise medically suitable to receive one.

### 2.2 Shared Decision‐Making

Shared decision‐making is a collaborative process that enables patients and healthcare professionals to make informed choices about treatment based on individual preferences, values and clinical evidence (NICE 2021). The UK's ‘No Decision About Me Without Me’ policy underscores the importance of patient autonomy in medical decision‐making, recognising that preferences may evolve over time (NICE 2011).

National and international guidelines recommend shared decision‐making as a critical component of kidney transplant decision‐making to ensure patients understand the risks, benefits and alternatives before making a treatment choice (KDIGO 2020). Studies suggest many patients with kidney failure have limited engagement in the decision‐making process, particularly those on in centre haemodialysis (UKKA and Kidney Care UK 2024). Decision aids aim to enhance patient understanding of available treatment choices (Elwyn et al. 2012). The kidney‐specific online tool Kidney Transplant Decision Aid (srtr.org) (Scientific Registry of Transplant Recipients, n.d.) can be used to support patients in comparing transplantation with dialysis. However, the primary focus of the tool is to encourage transplantation rather than support those who choose not to proceed.

### 2.3 Study Context

In 2016, a renal audit by clinicians in North Wales documented lower transplantation uptake compared to the national average and policy targets. The reasons behind this were unclear, but there was an assumption that the issue could be addressed through targeted interventions. The lack of patient perspectives highlighted the need for research into why some people chose not to pursue kidney transplantation.

**FIGURE 1:**
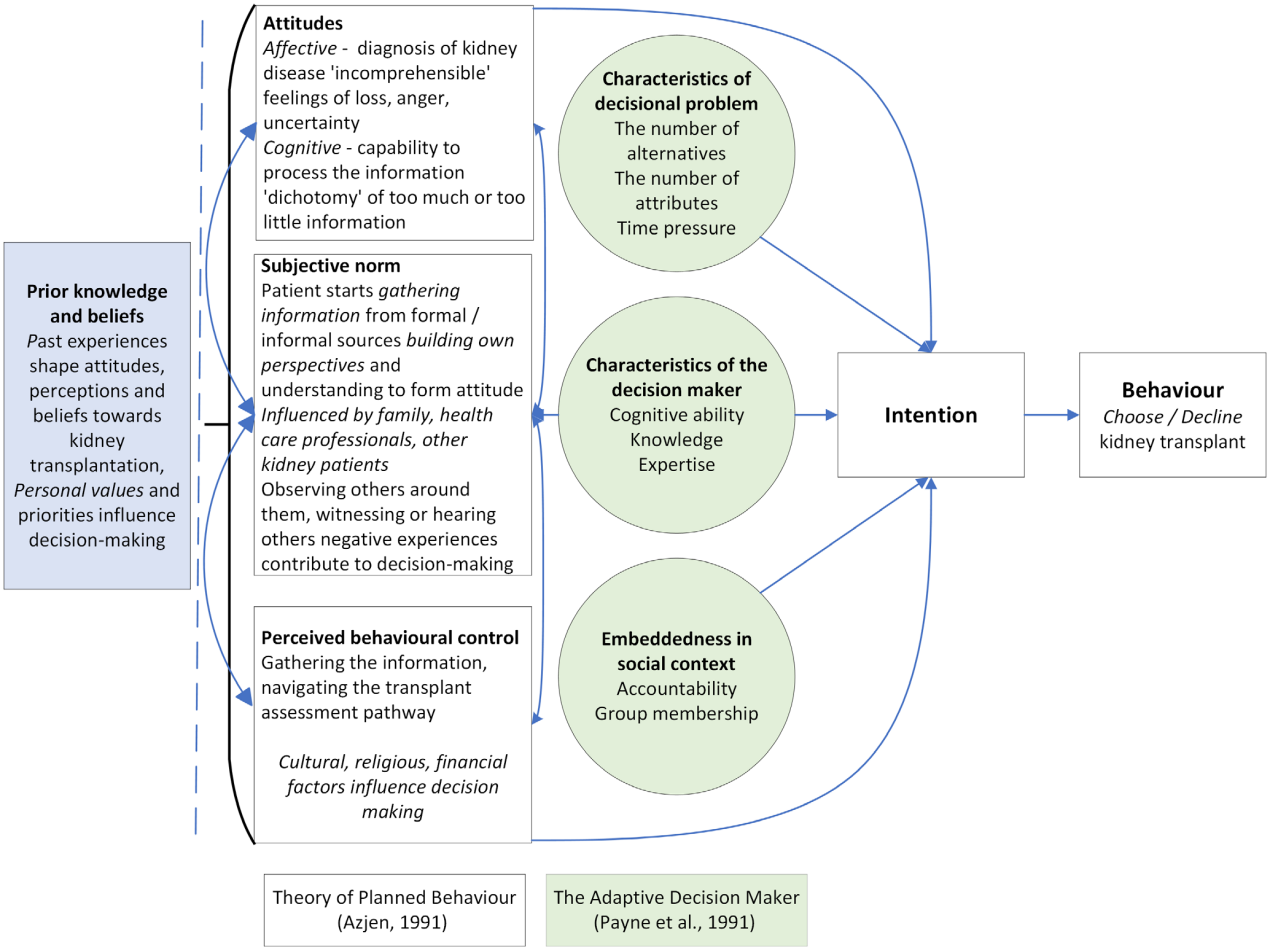
Enhanced theoretical model of the theory of planned behaviour and the adaptive decision maker framework developed from the QES (Jones et al. 2022).

The primary research reported in this paper builds on an initial qualitative evidence synthesis (QES) that explored the complexities of kidney transplant decision‐making (Jones et al. 2022). The QES identified six key themes, including patients' readiness to pursue kidney transplantation, access to information, navigating the assessment process, desire for or opposition to kidney transplantation, and uncertainties while waiting for a kidney transplant. A new enhanced theoretical model was developed, integrating the Theory of Planned Behaviour (Ajzen 1991) and the Adaptive Decision Maker Framework (Payne et al. 1993) (Figure 1) to better conceptualise the factors influencing patients' choices. Findings showed that decision‐making was highly individual and shaped by personal experiences, knowledge and perceived control. We concluded that further research was needed to understand why some individuals declined kidney transplantation, and to inform the development of personalised interventions and support tools to facilitate better informed decision‐making.

## 3 The Study

### 3.1 Aim

To develop an in‐depth understanding of people's perceptions and experiences of decision‐making and the reasons why they declined the opportunity of a kidney transplant.

## 4 Methods

### 4.1 Study Design

An interpretative phenomenological analysis (IPA) (Smith et al. 2009) ideographic approach was selected as the most suitable method to understand and explore individual experiences and perspectives (individual case) of people who said ‘no’ to a kidney transplant before going on to analyse data at group level using cross‐case thematic analysis.

### 4.2 Theoretical Framework

The enhanced theoretical model was used as an interpretive lens to analyse and understand how people with kidney failure made decisions about kidney transplantation (Figure 1).

### 4.3 Study Setting and Recruitment

Three Welsh National Health Service (NHS) Health Boards were selected, along with three purposively selected NHS Trusts in England to fill gaps in the purposive sampling frame, including a major teaching hospital with a more ethnically diverse patient population. NHS Health Boards (Wales) and NHS Trusts (England) provide comprehensive kidney transplant services.

The target sample size was 30, which is the maximum recommended for an IPA (Eatough and Smith 2017) reflecting the high homogeneity within the population and the need for maximum variation sampling to capture diverse perspectives on declining kidney transplantation. This ensured sufficient rich data to understand the phenomenon as experienced by people living with kidney failure (see File S1).

Recruitment was facilitated by kidney healthcare teams who distributed information packs to people who met the study inclusion criteria; recruitment posters were displayed in kidney units. Kidney charities and social media also advertised the study. All materials were available in Welsh and English, and interviews were offered in both languages, though all participants chose English.

A total of 69 people were contacted by kidney teams; 44 declined to participate (see File S2 for reasons for declining participation). An additional eight people contacted the primary researcher directly, but five did not meet the inclusion criteria and were excluded. Three others expressed interest in participating via email and were excluded, following concerns that they were not who they purported to be (Ridge et al. 2023). A £25 high‐street voucher was offered to those who were interviewed. Partners, family and carers were not recruited.

### 4.4 Inclusion Criteria

Individuals aged 18 years and older with kidney failure (Stage 4 or 5 eGFR 29 mL/min/1.73 m^2^), requiring or not requiring kidney replacement therapy, including individuals with failing kidney transplants.

### 4.5 Data Collection

Interviews were conducted by the primary researcher between August 2022 and June 2023. Participants were offered the choice of being interviewed face‐to‐face (at home, in a renal unit, or another comfortable location), by telephone, or virtually via Zoom or Microsoft Teams. Participants had full control over the timing, day and location of the interview.

Interviews followed a flexible, exploratory topic guide informed by the findings of the QES (Jones et al. 2022) and patient and public involvement (PPI) input (see File S3 for details of the interview guide sent to participants prior to the interview). The interview guide was further developed and evolved to explore and address emerging gaps and areas that lacked sufficient understanding. Interviews began with demographic data collection, including gender, age, marital status, ethnicity, educational attainment, and details of kidney care history. The first interview question asked participants about their experiences of having kidney disease. Additional prompts and questions were tailored to each individual. Probing questions were used, such as ‘can you tell me more about that?’ Interview questions allowed people to articulate their personal reasons for not wanting a kidney transplant.

Interviews varied in length, ranging from 19 min to 1 h 50 min, with virtual interviews generally being shorter. Participants were recruited and interviewed until no new themes or concepts emerged.

All interviews were digitally recorded and securely stored at Bangor University and transcribed by E.J. or a third‐party transcription service where confidentiality agreements were in place, and imported into NVivo (Version 11) for analysis.

### 4.6 Data Analysis

Data analysis followed the principles of IPA (Smith et al. 2009) and drew on the theoretical framework as an interpretive lens. All authors read the transcripts. Initial noting focused on content, language and participant meaning making. Initial coding was undertaken by the primary researcher; all authors checked selected transcripts for coding. Findings were discussed in weekly meetings to support the development of findings. The idiographic process privileged personal stories, experiences and kidney journeys. Kidney transplant decisions had taken place over many years.

Using hermeneutics, each transcript was analysed individually, paying attention to the person's unique perspective before moving on to cross‐case analysis and theme development. The data analysis process involved constructing a compelling and unfolding narrative to tell a coherent story and provide analytic commentary on the reasons and decisions attached to why they had declined a kidney transplant (Smith et al. 2009; Smith and Osborn 2008). The process was iterative, moving back and forth between reviewing codes, participant meanings and evolving interpretations. Emerging themes were identified, connections across themes explored and patterns across cases examined. Themes were subsequently collapsed, restructured and merged to provide deeper analysis and understanding of people's perceptions of kidney transplantation. Ongoing analysis maintained an idiographic focus, ensuring individual voices were preserved while identifying shared experiences in kidney transplant decision‐making. Participants were given ethnically sensitive pseudonyms.

### 4.7 Ethical Considerations

Ethical approval was obtained from the School of Medical and Health Sciences, Bangor University Research Ethics Committee (reference: 2022‐17,081), the South‐East Scotland Research Ethics Committee 01 (SES REC 1) on 16 May 2022, and the NHS Health Research Authority and Health Care Research Wales.

Informed written consent was obtained, a distress protocol was in place, and participants were, if appropriate, signposted to sources of additional support. Participants were informed that participation was confidential and would not affect their healthcare. They were advised that they could withdraw at any time without giving a reason and that any data collected before withdrawal would still be included in the analysis.

### 4.8 Rigour and Reflexivity

Reflexivity was essential to maintaining awareness of the primary researcher's background as a transplant nurse specialist and potential preconceptions. Co‐researchers brought expertise in nursing research, health services research and renal clinical psychology.

To minimise bias, reflexive journaling was maintained throughout data collection and analysis to document assumptions and stay engaged with participants' narratives. Regular meetings and detailed reflexive notes on recruitment, interviews and data interpretation ensured a transparent and rigorous analytical process. The co‐researcher MDT input further strengthened the approach.

Prior to commencing interviews, the primary researcher took time to establish a rapport with the participants; five people were known to her and were subsequently made aware of her new role as a researcher at Bangor University.

## 5 Findings

### 5.1 Characteristics of Participants

Thirty participants were interviewed, 19 males and 11 females aged 34 to 83 years (Table 1). No participants withdrew.

**TABLE 1:** Participant characteristics.

Participant	Age	Kidney replacement	Where they opted out of kidney transplant pathway
	Sex	Stage kidney disease	Main reasons for declining kidney transplant
	Ethnicity	Time on dialysis	
	Marital status		
P1 Philip	70s, male, white, married	Satellite unit HD 6 years	Declined to be worked up to be assessed for transplant eligibility. Too old for a kidney transplant, perception of kidney transplant too stressful, dialysis going well
P2 William	70s, male, white, married	Satellite Unit HD 5 years	Had investigations, seen by transplant team, declined to be listed. Medical mistrust, not enough information for his age group to weigh risks and benefits, too old
P3 Alyn	70s, male, white, widowed	Not on dialysis CKD 4 (eGFR 18)	Declined to be worked up to be assessed for transplant eligibility. Too old, denial, does not want to consider any KRT
P4 Peter	60s, male, white, separated	In centre HD 2 years	Declined to be worked up to be assessed for transplant eligibility. Dialysis going well, not ill or restricted by dialysis, previous experiences affect decisions
P5 Gareth	60s, male, white, separated	Nocturnal home HD 4.5 years	Removed themselves from the transplant waiting list. Previous trauma, doing well on Nocturnal Home HD, transplant centre too far
P6 Adam	40s, male, white, separated	Satellite unit HD 20 years (2 failed Tx)	Previously transplanted and declined another transplant. Previous transplants failed traumatic experiences
P7 Dylan	30s, male, white, single	Satellite unit HD 6‐7 years	Removed themselves from the transplant waiting list. Declined an offer of a kidney transplant when called by transplant centre. Fear transplant, unable to have time from employment
P8 Tomos	40s, male, white, single	In centre HD 6 years	Declined to be worked up to be assessed for transplant eligibility. Dialysis going well, transplant will not improve quality of life
P9 Hannah	30s, female, white, partner	Satellite unit HD 16 years	Declined to be worked up to be assessed for eligibility. Dialysis going well, previous traumatic experience led to mistrust in kidney doctors, does not want someone else's kidney
P10 George	70s, male, white, married	Satellite unit HD 6 months	Had investigations locally, declined to be referred to transplant team. Too old, prefer younger people to be transplanted, dialysis going well
P11 Lynne	60s, female, white, separated	Not on dialysis CKD 5 (eGFR 13)	Agreed to be referred to transplant team, cancelled appointment. Not on dialysis, too old, when times comes dialysis preferred treatment, prefer younger people to be transplanted
P12 Eifion	60s, male, white, widowed	In centre HD 2 years	Agreed to be referred to transplant team, cancelled appointment. Dialysis going well, uncertain if transplant will improve quality of life, transplant centre too far
P13 Jim	60s, male, white, married	Not on dialysis CKD 4 (eGFR 18)	Declined to be worked up to be assessed for eligibility. Post traumatic stress, does not want KRT, too old, does not want someone else's kidney
P14 Mark	50s, male, white, married	Nocturnal home HD 6 years	Removed themselves from the transplant waiting list. Nocturnal Home HD going well, quality of life as good as transplant, fear recurrence IGA, does not want someone else's kidney
P15 Helen	50s, female, white, married	Home HD 5 years	Declined to be worked up to be assessed for transplant eligibility Home dialysis going well, previous traumatic experience, fear of surgery
P16 Sadiya	70s, female, ethnic minority, married	Not on dialysis CKD 5 (eGFR 11)	Removed themselves from the transplant waiting list. Declined an offer of a kidney transplant when called by transplant centre. Too old, prefer someone younger to be transplanted, not on dialysis yet
P17 Sean	40s, male, white, married	Nocturnal home HD 23 years (2failed Tx)	Previously transplanted and declined another transplant. Previous transplants failed traumatic experiences
P18 Gerraint	70s, male, white, married	Training Nocturnal home HD 8 months	Declined to be worked up to be assessed for transplant eligibility. Previous cancer, concerned risk of cancer post‐transplant, feeling well on dialysis
P19 Bethan	60s, female, white, single	In centre HD 2.5 years	Declined to be worked up to be assessed for eligibility. Previous traumatic experiences, fear of transplant surgery
P20 Richard	70s, male, white, married	CAPD 3 months	Withdrawn and disengaged from the kidney transplant assessment work‐up process. Too old, dialysis going well
P21 Gail	60s, female, white, separated	Pre CKD 4	Declined to be worked up to be assessed for transplant eligibility. Previous traumatic experiences, mistrust healthcare teams, fear of surgery and recovery
P22 Owain	40s, male, white, married	In centre HD 27 years	Declined to be worked up to be assessed for transplant eligibility. Dialysis going well, previous traumatic experience led to mistrust in kidney doctors
P23 Harry	60s, male, white, married	Home HD 8 years	Declined to be worked up to be assessed for transplant eligibility. Mistrust in kidney doctors. Home dialysis going well and as good as a transplant. Fear risk of immunosuppression, risk of infection. Previous negative experiences of healthcare
P24 Gloria	60s, female, white, married	APD 2 years	Declined to be worked up to be assessed for transplant eligibility. Too old, quality of life good, fear risk of increased immunosuppression, prefer someone younger to be transplanted
P25 Martin	70s, male, white, widowed	In centre HD 8 years	Removed themselves from the transplant waiting list after not receiving transplant. Transplantation is a gamble, fear transplant would leave him worse off, too old prefer someone younger to be transplanted
P26 Jean	80s, female, white, separated	APD 5 years	Removed themselves from the transplant waiting list after not receiving transplant. Too old, prefer someone younger to be transplanted, good quality of life unrestricted on APD
P27 Karen	60s, female, white, partner	Satellite unit HD 5 years	Declined to be worked up to be assessed for transplant eligibility. Does not want someone else's kidney, no guarantee a transplant would work, does not want side effects from immunosuppression, prefer someone else to be transplanted
P28 Jacqui	60s, female, white, married	APD 11 months	Withdrawn and disengaged from the kidney transplant assessment work‐up process. Fear of surgery, fear of hospital, still coming to terms with diagnosis, previous traumatic experiences of healthcare, does not feel she deserves a transplant
P29 Fred	80s, male, white, married	Satellite unit HD 7 years	Had investigations, seen by transplant team, declined to be listed. Risk of surgery at old age, someone younger to be transplanted, previous traumatic experiences
P30 Soma	30s, female, British South Asian, separated	In centre HD self care 19 years (1failed Tx)	Removed themselves from the transplant waiting list. Declined an offer of a kidney transplant when called by transplant centre. Previously transplanted and declined another transplant. Previous transplant failed, traumatic experiences whilst transplanted

Abbreviations: APD, automated peritoneal dialysis; CAPD, continuous ambulatory peritoneal dialysis; CKD, chronic kidney disease; DD, deceased donor; HD, haemodialysis; KRT, kidney replacement therapy; LD, living donor; Tx, transplant.

### 5.2 Individual Stories

The last column in Table 1 reports the main reasons why individuals said ‘no’ to a kidney transplant.

### 5.3 Point of Decision‐Making on the Kidney Transplant Clinical Pathway

**FIGURE 2:**
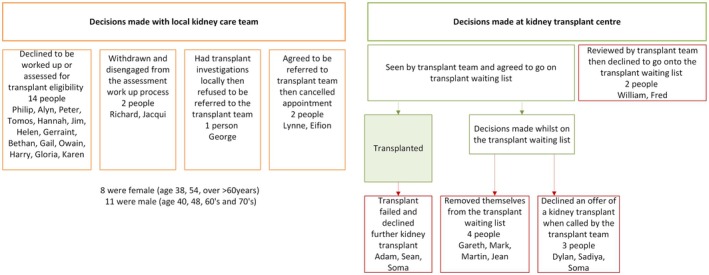
Point of decision‐making on the kidney transplant pathway.

The decision not to have a kidney transplant was made by people at various points along the kidney transplant clinical pathway and are identified in Figure 2. Everyone described having received kidney transplant education from their kidney teams (usually nurse led), albeit at different stages in their kidney journey. They were knowledgeable about kidney transplantation as a kidney replacement option. Almost all were known by their kidney healthcare teams for more than a year before needing kidney replacement therapy and had time to contemplate and make treatment decisions. Those who had slow kidney disease progression had time to consider what kidney treatment would work best for them. People who needed to begin dialysis as an emergency had no time to make treatment decisions.

### 5.4 The Overarching Picture Concerning Kidney Transplant Decision‐Making

The decision to decline a kidney transplant was, for the majority, a concrete decision that was complex and multifaceted. People explained that there was no single, simple reason for their choice; rather, multiple reasons were shared, which transcended several themes. Their decisions not to pursue a kidney transplant (or another transplant) appeared to be carefully thought through, and people viewed it as the right choice for them.

**FIGURE 3:**
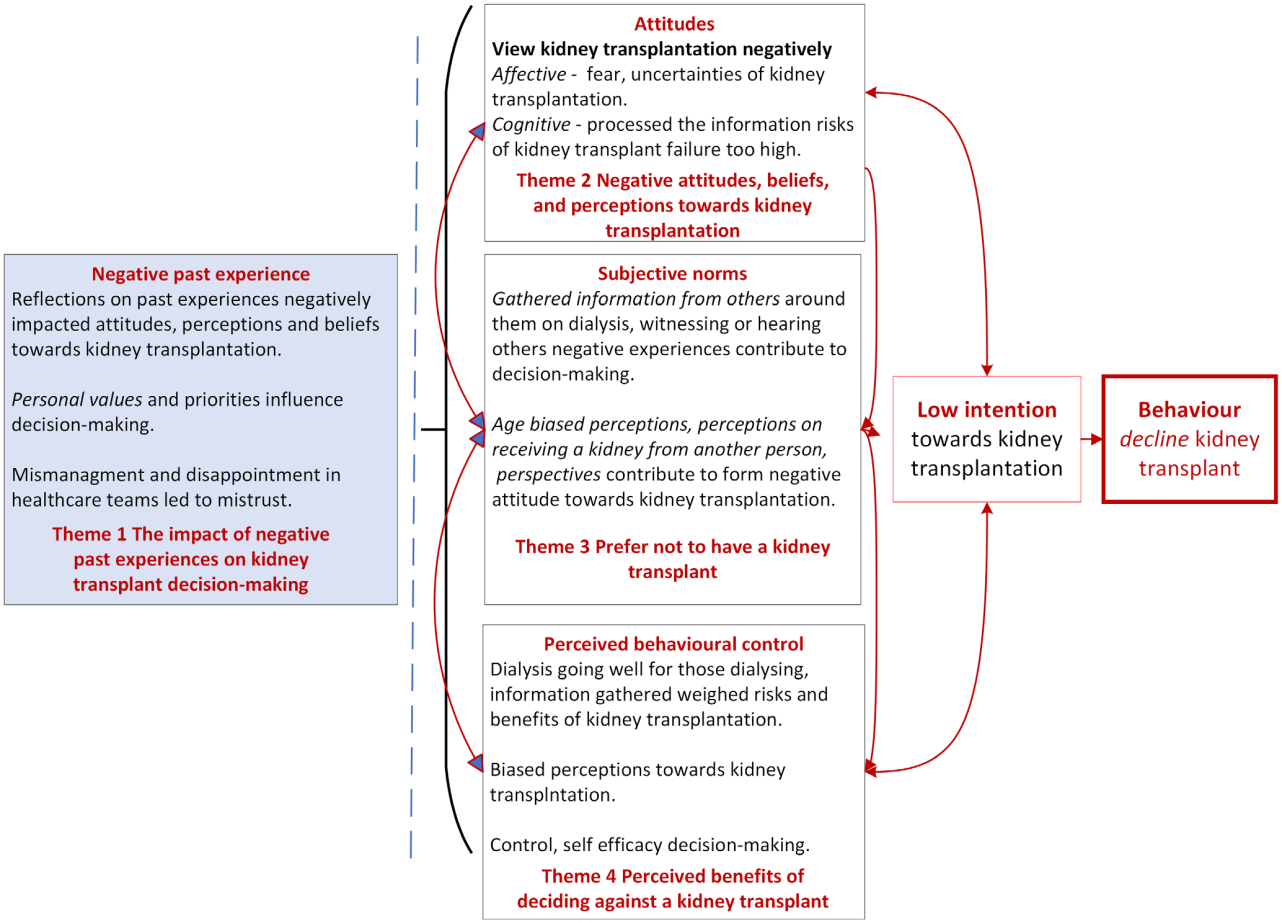
Conceptual relationship between each Theme and Theory of Planned Behaviour.

A visual representation (Figure 3) offers an overview of the interconnected themes and subthemes, illustrating their conceptual relationships. The following findings prioritise individual narratives, reporting the unique idiographic experiences of people before drawing connections at the group level. These connections are then mapped against theoretical frameworks to enhance understanding.

To preserve the integrity of people's stories, words, metaphors and concepts from their interviews were incorporated into theme titles. The themes were highly interconnected, providing insight into the lived experiences of individuals with kidney failure.

### 5.5 Themes and Subthemes

Four themes were developed that explain the experiences, decisions and meanings of group and individual stories. Each of the four themes had subthemes and is summarised in Table 2.

**TABLE 2:** Themes and subthemes.

Theme	Subtheme
Theme 1 The impact of negative past experiences on kidney transplant decision‐making	Reflections on experiences of kidney disease diagnosis Traumatic experiences of beginning dialysis as an emergency Experiences, recollections and personal impact of the COVID‐19 pandemic Kidney transplant failure Negative experiences of immunosuppressant medication Experiences of infection leading to failed dialysis access
Theme 2 Negative attitudes, beliefs and perceptions towards kidney transplantation	Perceptions and experiences of kidney transplant failure ‘Why take the risk of being worse off?’ Fear of increased cancer risk following kidney transplantation Fear of post‐transplant diabetes Perceptions of being too old for a kidney transplant Younger adults perceived they were too old for a kidney transplant Fears, attitudes and beliefs about receiving a kidney from another person Negative perceptions of living kidney transplantation
Theme 3 Prefer not to have a kidney transplant	Preferring someone younger to be transplanted Deciding not to have a kidney transplant ‘They presumed I wanted to have a kidney transplant’ shared decision‐making conversations? ‘No…I do not want a transplant, what do you not understand?’ conversations about decisions with family and kidney teams ‘Statistics are not encouraging’ weighing up the evidence Decisions to come off the kidney transplant waiting list
Theme 4 Perceived benefits of deciding against a kidney transplant	Delaying decision‐making ‘Do not need to decide yet as I am not on dialysis’ Dialysis experiences preferring to remain on dialysis in preference of kidney transplantation Home dialysis having similar outcomes to transplantation

### 5.6 Theme 1: The Impact of Negative Past Experiences on Kidney Transplant Decision‐Making

Past experiences significantly shaped individuals' attitudes towards kidney replacement and transplant options. All participants had chosen not to have, or not to have another kidney transplant, often conflicting with the views of family members and healthcare teams. Conversations involved loved ones—spouses, parents, children, friends—and healthcare professionals such as doctors, nurses, social workers and psychologists. People reflected on the emotional toll of being diagnosed with kidney disease and adjusting to dialysis, especially when started unexpectedly or as an emergency. Those who previously saw themselves as ‘healthy’ found this particularly distressing. Past traumatic healthcare experiences often led to negative perceptions of transplantation. Individuals like Adam, Sean and Soma, who had experienced failed transplants, were hesitant to try again due to the lasting emotional, mental and physical scars.

The COVID‐19 pandemic also negatively influenced decisions. Some experienced delays in diagnosis, while others adapted to starting dialysis during this period. With transplantation paused, people had more time to consider their options.

Past experiences carried specific meanings: childhood diagnosis or adult onset often signified a loss of normal life trajectory, like a peaceful retirement. Negative attitudes towards healthcare teams frequently stemmed from past mistrust and perceived poor care, impacting views on transplantation and healthcare relationships.

#### 5.6.1 Reflections on Experiences of Kidney Disease Diagnosis

Diagnosis experiences varied by age. Some were diagnosed as children or teenagers, others as adults. Regardless of timing, past experiences influenced attitudes towards treatment, especially transplantation. Many felt let down by healthcare teams, citing delayed diagnoses, poor follow‐up, and medication‐related kidney damage, fostering mistrust and leading some to reject transplantation.

##### 5.6.1.1 Diagnosis as an Older Adult

Seventeen people were diagnosed in their 50s, 60s, or 70s, often through routine checks or unrelated investigations. The lack of symptoms made the diagnosis shocking, disrupting life and retirement plans. Sadiya, diagnosed at 60, described feeling devastated:

My kidney function was bad, I was devastated. But I was quite young, I'd just retired, I was looking forward to my life, and I thought that was the end of my life… I said oh no, I would rather die [than be on dialysis] … that was my first reaction.

Others felt frustration and resentment, struggling to accept their new reality after feeling well before diagnosis.

##### 5.6.1.2 Blaming Medication for Kidney Damage

Jim and William attributed their kidney failure to prescribed medications without adequate monitoring, feeling the condition was avoidable. William linked his failure to frequent hospitalisations and medication:

I thought, I'm not surprised, with all this stuff [medications] I've been taking. And I ended up on dialysis.

##### 5.6.1.3 Disappointment With Follow‐Up Care

Fred, Gail, Jim and Hannah expressed disappointment with healthcare professionals for not providing a timely follow‐up. Gail, who had a nephrectomy, only learned of her kidney failure years later during a routine GP blood test. Fred recalled being told his kidney function needed checking, but a follow‐up never happened:

They never did keep an eye on it [kidney function], I was never called back to have it checked again, and I didn't volunteer, and then I just became really poorly.

Such experiences fostered mistrust and shaped decisions to decline transplantation.

##### 5.6.1.4 Experience of Suffering and Self‐Blame

Long‐term suffering and negative healthcare interactions reinforced distrust. Jacqui, diagnosed with Type 2 diabetes in her early 50s, felt blamed for her kidney disease:

I didn't deserve it [a transplant] … because so many of the nurses had made it feel like it's your fault [having diabetes] … so I didn't think I deserved it [a transplant].

For some, these experiences solidified their belief that transplantation was not the right option.

##### 5.6.1.5 Inevitable Kidney Failure Decline

Richard, Harry and George had inherited polycystic kidney disease (PKD). They expressed frustration at the inevitability of kidney failure despite monitoring and lifestyle changes. Harry felt powerless despite tracking his kidney function:

They weren't doing anything that could protect the kidneys … it was just a case of we can't do anything, they're going to fail.

After seeing his mother die post‐transplant, Harry sought private care for better management, only to hear the same prognosis, deepening his sense of helplessness.

##### 5.6.1.6 Diagnosis as a Child

Those diagnosed as children or young adults (e.g., Adam, Dylan, Tomos, Hannah, Sean, Owain, Soma) often faced misdiagnoses, delayed recognition and exclusion from decision‐making. Symptoms were sometimes misinterpreted, like Soma's teenage weight loss, dismissed as a phase. Social and educational challenges shaped their identities and future expectations.

Soma, Owain and Tomos struggled with being left out of treatment choices. Soma, a British South Asian woman, felt unprepared for dialysis as decisions were made for her by her parents and kidney team:

I didn't know what my arm was for, I didn't know what the fistula was for, but I was ready for dialysis.

Traumatic medical experiences during childhood shaped fears around transplantation. Owain, who had severe incontinence, feared self‐catheterisation post‐transplant due to a painful experience:

A nurse tried to take the catheter out without deflating the balloon … that trauma has stuck with me.

Despite hardships, some found resilience, like Owain, who felt his experiences shaped his strength and identity.

#### 5.6.2 Traumatic Experiences of Beginning Dialysis as an Emergency

Dylan, Eifion, Helen, Jean, Karen and Fred began dialysis as an emergency. With no prior knowledge of kidney disease, their experiences were marked by shock and fear.

Karen recalled being terrified when she was told she needed a neckline catheter for emergency dialysis:

They said, we're just going to take you through to the operating theatre and put a line in. And I thought what the hell is dialysis?

Karen felt powerless, describing treatment as being ‘done to them’ with little explanation or emotional support.

#### 5.6.3 Experiences, Recollections and Personal Impact of COVID‐19

Eight people, Peter, George, Eifion, Gerraint, Bethan, Richard, Gloria and Jacqui, were diagnosed or began dialysis during the COVID‐19 pandemic, which described appointment delays and a lack of face‐to‐face education about kidney replacement options.

Gloria, who had lupus nephritis, was told she needed to see a nephrologist urgently. However, due to COVID‐19, she was told: ‘We're not taking on new patients until things settle down … you're in the queue’. By the time she was seen by a nephrologist, irreversible kidney damage had already occurred.

#### 5.6.4 Kidney Transplant Failure

Adam, Sean and Soma, who had previously received kidney transplants, described traumatic experiences leading to their decision to decline another transplant. Adam, who received a living donor transplant, nearly died after contracting Pneumocystis pneumonia (PCP). Adam was left devastated when his second transplant failed after complications:

I had to have three operations within a week … I nearly lost my life.

Similarly, Sean's two transplants failed due to recurrence of Focal Segmental Glomerulosclerosis (FSGS) a rare disease which causes scarring to kidney glomeruli. He calculated that his chance of FSGS recurrence was over 50%, making him unwilling to risk another transplant.

I knew that if I do have another transplant, the likelihood is it will recur in a third transplant.

Soma, who received a transplant at age 18, described feeling completely unprepared and uninformed, likening the experience to a broken fairy tale:

All I heard was transplant, and I thought it was my happily ever after … but I found out the hard way that it was not [her transplant failed].

#### 5.6.5 Negative Experiences of Immunosuppressant Medication

Immunosuppressive medication, essential for preventing kidney transplant rejection and managing autoimmune conditions, was widely rejected by people due to its severe side effects. Several people had previously taken immunosuppressants either following a kidney transplant or for conditions such as vasculitis, lupus, Immunoglobulin A (IgA) nephropathy, and FSGS. Their negative experiences with these medications led to a strong reluctance to take them again.

Older people, including William, Gloria, Martin and Jean, were especially concerned about the potential impact of immunosuppressants on their already fragile health. The risk‐to‐benefit ratio was seen as unacceptable, as the medication had previously caused them significant illness and distress.

#### 5.6.6 Experiences of Infection Leading to Failed Dialysis Access

People with kidney failure rely on dialysis access to undergo kidney replacement treatment. Failure of dialysis access forced William, Tomos, Lynne, Eifion, Mark and Bethan to re‐evaluate their treatment options, leading to uncertainty and distress. Bethan, having faced repeated complications and infections, doubted the success of any future medical interventions. This uncertainty extended to kidney transplantation:

If I had the transplant and something happened, you know, went wrong.

The emotional toll of repeated dialysis failures shaped perceptions of transplant risks, reinforcing concerns that a kidney transplant might not provide the stability they sought.

### 5.7 Theme 2: Negative Attitudes, Beliefs and Perceptions Towards Kidney Transplantation

Building on Theme 1, past negative experiences shaped negative attitudes towards kidney transplantation. Fear of transplant failure was common, regardless of age, gender, or ethnicity. People worried that a failed transplant would leave them worse off—those on dialysis feared returning to it, while those not yet on dialysis feared starting sooner. Previous transplant failures discouraged Soma, Adam and Sean from retrying.

Concerns included fears of dying, developing cancer, post‐transplant diabetes, or being too old. Deceased donor kidneys raised anxieties about the donor's medical history, while living donation prompted fears of pressure and potential harm to loved ones. These perceptions, rooted in past experiences, reinforced the belief that transplantation was too risky, with dialysis seen as a more stable, predictable option. William compared ‘living a good 10 years on dialysis’ to a ‘miserable 15 years’ post‐transplant.

#### 5.7.1 Perceptions and Experiences of Kidney Transplant Failure ‘Why Take the Risk of Being Worse Off?’

Fear of transplant failure was a dominant concern. Some, like Soma, Adam and Sean, feared failure based on personal experience, while others, like Tomos, Mark and Owain, internalised the belief that transplants were unlikely to succeed. Owain expressed doubts:

I just have a feeling that you could have one [a transplant] and it go in a week, a month, it could go in a year.

Dialysis patients who had witnessed multiple transplant failures reinforced the belief that transplantation was not a permanent solution. Tomos reflected:

If I have [a kidney transplant], how long will it last? Some people have had kidneys for 15, 20 years. Some don't last three months, and [they are] back to dialysis.

The fear of failure made transplantation emotionally distressing. Gareth feared returning to dialysis shortly after a transplant:

Oh, imagine having a [kidney] transplant now, and having to go back on dialysis after six weeks… that would finish me.

##### 5.7.1.1 Fear of Dying After a Kidney Transplant

Owain, Bethan, George, Philip, William, Tomos, Helen, Richard, Gloria and Eifion feared dying during or after transplant surgery. Owain described knowing dialysis patients die after transplantation, shaping his negative view:

I've seen a lot of people have transplants, and unfortunately, I've seen a lot of people die after having transplants… you think to yourself, was that the transplant that did that?

Bethan was similarly affected by a friend's death shortly after a transplant:

This friend of mine died… he had a transplant in November, and he died in February. It put me off altogether.

Helen, having experienced intensive care before, feared not waking up from surgery:

The thought of having to go into a [kidney transplant] operation, and to be put to sleep, thinking you're not going to wake up… I just can't do it.

##### 5.7.1.2 Negative Experiences of Hospitalisation

Traumatic hospital experiences also influenced negative views on transplantation. Gail described a frightening emergency surgery experience:

They put the mask over my face, and I just panicked… they [theatre staff] were still fooling around.

Inadequate post‐operative care deepened her fear of future procedures, including transplantation, and she worried about the lack of support:

…who's going to look after me?

##### 5.7.1.3 Kidney Transplantation Does Not Cure Kidney Disease

A common belief was that transplantation does not cure kidney disease but is just an alternative to dialysis and questions its long‐term effectiveness, William reflected:

People who are saying ‘no’ must have given it some thought…. [A transplant] is not the panacea; transplant is not a cure.

Richard, Sadiya, Jim, Tomos, William, Hannah, Karen, Owain, Helen, Phillip, Gareth, Eifion and Bethan, viewed transplantation as just another treatment rather than a permanent solution, opting for dialysis as the more predictable option.

##### 5.7.1.4 Perceived Burden and Upheaval After a Kidney Transplant

The perceived burden of post‐transplant care was a significant concern. Frequent hospital visits after transplantation were seen as a strain on individuals and families. George described the impact:

There's all the upheaval, going backwards and forwards to the [transplant centre] and if it does not work, I could have been made worse.

For George, Bethan and Gail, the uncertainty and potential disruption made the stability of dialysis more appealing. Adam, Sean and Owain had young children and were concerned they would burden their family if they were to be transplanted.

#### 5.7.2 Fear of Increased Cancer Risk After Transplantation

People feared the increased cancer risk from immunosuppressants, especially those who had survived cancer, like Phillip, George, Gerraint and Gloria. Gerraint, a bladder cancer survivor, was particularly cautious:

I wouldn't wish to be exposed to a greater risk of getting cancer… I don't particularly want an increased susceptibility to other cancers.

For these individuals, the cancer risk outweighed the potential benefits of transplantation.

##### 5.7.2.1 Fears of Increased Risk of Skin Cancer

Mark, Lynne and Harry feared how immunosuppressants might limit their lifestyle. Harry worried about constant sun protection:

They said, ‘you're going to have to wear a hat in the summer all the time.’ That's not freedom to travel… I would have lived a life of fear on transplant.

George shared his brother's experience of post‐transplant skin cancer:

He's [brother] had a big piece taken out of his ear, a big hole in his foot, a big cancer on his face… He thought he was going to lose his jaw.

These fears reinforced the belief that dialysis was safer and more predictable.

#### 5.7.3 Fear of Post‐Transplant Diabetes

People were also concerned about the risk of post‐transplant diabetes. Sean and Owain, though not diabetic, feared potential impacts:

The risks [following a kidney transplant] outweigh [the benefits]—there are risks of diabetes, risks of infections, it's just not worth it. (Sean)

Sadiya, with a family history of diabetes, worried she was at increased risk, making transplantation seem too uncertain.

#### 5.7.4 Perceptions of Being Too Old for a Kidney Transplant

Older people (60+) often feel too old for transplantation, preferring dialysis as a familiar, stable option. Martin, in his 70s, reasoned:

A transplant is a gamble, you could be worse off. Dialysis is better—the devil you know.

Philip, aged 70, questioned the effort of transplantation if it failed:

Say if it didn't work, well then, you'd have to go through a lot more again… why the hell did you bother?

Many reflected on their remaining years, feeling they had already lived full lives. Sadiya recalled her consultant's advice:

…[A kidney transplant] won't prolong your life. It may give you a slightly better quality of life, but it won't prolong it.

Even younger adults, like Hannah and Soma in their 30s, felt they were already too old, seeing dialysis as a safer option.

#### 5.7.5 Fears, Attitudes and Beliefs About Receiving a Kidney From Another Person

Hannah, Mark, Sean, Jean and Karen felt uneasy about receiving an organ from another person, fearing disease recurrence. Mark, with IgA nephropathy, was aware of the risk:

There's always a chance that the IgA [which destroyed my kidneys] could well destroy a transplanted kidney.

Others disliked the idea of a foreign organ inside them. Mark expressed:

It's not like I'd become impure, but it just feels very different… I just cannot get my head around having that.

Hannah felt uneasy about someone dying for her to live:

I don't want anyone else's organ inside me …. Thinking of someone else who has had to die for you to be living, that's not me.

Spiritual and ethical concerns also shaped decisions. Although religion was not explicitly cited, spiritual beliefs influenced decisions for Karen. Karen, a believer in reincarnation, wanted to keep her body whole after death:

I want to be buried whole… because I believe in reincarnation, and you have to be whole to be reincarnated.

#### 5.7.6 Negative Perceptions of Living Kidney Transplantation

Some felt pressured to consider living donation, especially by medical teams. Martin, Jean and Fred considered it but didn't want to burden loved ones. Fred, 80, noted:

Because I didn't have a living donor, it would be at least two years, possibly more… I didn't have a living donor available.

Others feared potential harm to donors. Bethan, caring for her young grandson, refused her children's offers:

If I took a kidney off them, and something happened to them… they've got their lives to live.

William was adamant about protecting his son:

I wouldn't let him [my son] give me a kidney. He's got a young family… I'm just going over the hill.

For those with polycystic kidney disease, like George and Richard, using a family member as a donor was particularly problematic, given the risk that other relatives might need a kidney in the future.

### 5.8 Theme 3: Preferred Not to Have a Kidney Transplant

People shared personal stories about choosing not to pursue a kidney transplant, highlighting a complex emotional journey. Many had made their decision before starting dialysis, carefully weighing risks and benefits. Despite recognising transplantation as an option, they often felt their preferences were overlooked by family and healthcare teams.

#### 5.8.1 Preferring Someone Younger to Be Transplanted

Older people often felt that younger individuals deserved transplants more, as they had longer lives ahead and suffered more on dialysis. Twelve participants, including Phillip, Peter, George, Lynne, Eifion, Sadiya, Bethan and Fred, shared this belief:

There's a young guy there [on dialysis] who must be no more than 18, he deserves a transplant… People in their seventies and eighties… they don't really. (Fred)

Others felt relieved when younger people received transplants:

We often [talk] on the [dialysis] unit… I wish so and so could get one, because they're young. It's nice to see the youngsters getting them. (Karen)

Some, like Eifion and Jacqui, believed they didn't deserve a transplant. Eifion struggled to explain why:

I don't think I deserve it… Doctor [name] used to say to me, ‘Why do you think like that? Everybody's entitled to a transplant if they need it.’

Others, like Lynne, cancelled transplant assessments, not wanting to waste the surgeon's time:

I don't think I want a transplant, and I don't want to waste the doctor's time talking about it when he could be talking to somebody else.

#### 5.8.2 Deciding Not to Have a Transplant

For many, the decision not to pursue transplantation was final. Conversations with family and healthcare teams informed their choices, but most decided against it:

I think they [my family] understood, but it didn't stop them trying to harass me into getting one… But I don't even think about it. It's just a complete decision—100% decision—I'm not having one. (Peter)

Philip also faced family pressure:

They said, ‘you're having it done.’ I said, ‘no, I'm not’… I don't think I could put up with the mental strain of it.

#### 5.8.3 ‘They Presumed I Wanted to Have a Kidney Transplant’ – Shared Decision‐Making Conversations

Mark, Gail and Harry felt their kidney teams assumed they would want a transplant, often pushing them towards the decision without considering their preferences. Mark reflected:

Nobody ever asked me did I want a transplant… there's almost an expectation that you're going to go to transplant.

Hannah and Tomos felt their consultants dismissed their decision:

They keep on saying that you'd be better off, but I know a lot of people who have had transplants, and they haven't lasted that long. (Hannah)

#### 5.8.4 ‘No … I Do Not Want a Transplant, What Do You Not Understand?’ Conversations About Decisions With Family and Kidney Teams

Philip, Peter, Adam, Tomos, Hannah, Helen, Karen, Gerraint, Gloria, Sean, Soma, Dylan, Mark and Owain described being repeatedly questioned about their decision, causing frustration:

They just keep asking me… ‘Have you changed your mind yet?’ No, I don't want a transplant—what don't you understand? (Tomos)

This pressure sometimes led to suspicion about healthcare motives. Helen wondered if transplants were encouraged because they were cheaper than dialysis, while Karen felt pressured to free up dialysis space:

It was almost as if, could you please have a transplant so we can have the bed free?

Helen, Jacqui, Karen, Peter, Bethan and Soma were referred to psychologists after refusing a transplant, feeling misunderstood. Peter recalled:

They [dialysis nurses and doctors] did ask me if I wanted to see a psychologist once… I think they think I'm nuts… I'm not. I'm normal.

Soma felt coerced into a psychological assessment:

The doctor said, ‘Okay, I think you need to speak to a psychologist.’ I went, ‘I don't think I need to… But if it gets you off my back, book me in.

Harry recalled educational sessions that seemed biased towards transplantation, with little mention of dialysis. Harry, who preferred home dialysis, felt forced into transplant discussions:

They might as well have called it pre‐transplant, because they never mentioned dialysis at all.

Conversations with family were mixed, with some pushing for transplantation and others supportive. Despite family pressure, people remained firm in their decisions:

[My wife] would sooner have me here on dialysis than not at all. (George)

#### 5.8.5 ‘Statistics Are Not Encouraging’ – Weighing Up the Evidence

Despite gathering information about kidney transplantation, William, Richard, Harry and Gerraint felt they lacked personalised data relevant to their age and condition. They consulted kidney teams and medical journals but found statistics on transplantation insufficiently transparent.

Very few papers are based on concurrent data… technology changes so quickly, but it's almost out of date before you publish it. (Harry)

For William, the absence of tailored risk assessment influenced his decision:

I needed to know if I would be better with a transplant… The doctor said, ‘30% of people are OK, 30% have some problems, and 30% are a disaster.’ Well, which group am I in?

Without individualised data, people found it hard to judge whether transplantation would improve their quality of life compared to dialysis.

#### 5.8.6 Decisions to Come Off the Kidney Transplant Waiting List

Several people who had been on the transplant list, including Gareth, Mark, Dylan, Soma, Sadiya, Martin and Jean, ultimately chose to withdraw.

Gareth and Mark, who were on nocturnal home haemodialysis, felt well and found relief in their decision. Mark, who is methodical in decision‐making, reflected:

I'm a very cerebral person, I mull decisions over for weeks… When I left the transplant clinic, I felt relieved.

Dylan declined multiple transplant offers due to work commitments:

I've been asked to go for a transplant twice, I had to turn them down, because things weren't right at home.

Soma, having experienced transplant failure, found being put back on the list distressing. When called for an altruistic living donor transplant, she refused:

I've said no, please respect my decision. Why am I getting berated? I've been cut open, I've bled, I've cried, I can't do it anymore.

Reflecting later, she felt relieved, especially during the COVID‐19 pandemic:

Had I had that transplant… with COVID‐19 patients dying left, right, and centre, and me needing blood tests and biopsies—what would I have gone through?

##### 5.8.6.1 Feeling Too Old to Remain on the Transplant List

Sadiya, Martin and Jean initially wanted a transplant but reconsidered after being called and not selected, as they were worried about their age and health. Jean, in her 80s, had been called several times but was not chosen. She reflected:

By the time the list was reinstated [post‐COVID], I was not the woman I was [seven years earlier]. I just thought—no, I couldn't face a big operation.

Both Martin and Jean felt dialysis was the better option. Jean summed up her decision:

It's better the devil you know than the one you don't… I decided to stick with the devil I know.

##### 5.8.6.2 Difficulties Travelling to the Transplant Centre

Gareth, Dylan and Eifion cited transportation difficulties as a barrier to transplantation. Gareth and Dylan found the distance to the transplant centre challenging, while Eifion missed his assessment because he didn't know he could request hospital transport and didn't want to burden his daughter.

### 5.9 Theme 4 Perceived Benefits of Deciding Against a Kidney Transplant

This theme explored why individuals found kidney transplantation less appealing than dialysis. All participants had actively chosen not to have a transplant, believed they would not benefit, and felt positive about their decision.

Dialysis was seen as more familiar and manageable since people knew what it involved and were able to maintain a good quality of life. People of all ages and genders on dialysis shared positive experiences, described stable routines, independence and strong relationships with dialysis nurses. Some likened dialysis to a comfortable, safe environment, which allowed them to live a full life with fewer restrictions than a transplant might impose.

A key reason for rejecting transplantation was feeling well on dialysis. Those on dialysis described having energy and leading normal lives, reinforcing the idea that dialysis was ‘better the devil you know’. People on home dialysis reported comparable survival and quality of life outcomes between home dialysis and a cadaveric kidney transplant. For most, quality of life mattered more than longevity. They saw transplantation as a risk: ‘Why bother with a transplant when dialysis is working well?’

People also rejected the label of *‘kidney patient’*, resisting the idea that dialysis made them ill. Peter emphasised that choosing dialysis was an act of self‐efficacy—affirming control over their treatment choices:

Dialysis keeps me alive. Why change something that works?

#### 5.9.1 Delaying Decision‐Making: ‘Do Not Need to Decide Yet as I Am Not on Dialysis’

For those not yet on dialysis, including Alyn, Lynne, Jim, Sadiya and Gail, the decision about transplantation felt irrelevant. Stable kidney function and no symptoms meant they were not ready to think about treatment options.

Alyn, who had maintained stable kidney function for 16 years, shared his typical clinic interaction:

He'd ask, ‘How are you?’ and I'd say ‘Fine, unless you tell me different’.

For Alyn and Lynne, ignoring kidney disease and only considering one day at a time was a coping strategy:

You cannot predict the future, can you? How you are going to be, how your health is going to be, I do not think far beyond tomorrow. (Lynne)

#### 5.9.2 Dialysis Experiences—Preferring to Remain on Dialysis Rather Than Undergo Transplantation

Philip, Peter, Gareth, Adam, Tomos, Hannah, George, Eifion, Mark, Helen, Sean, Gerraint, Richard, Owain, Harry, Gloria, Jean, Karen and Soma had adjusted to dialysis and felt that transplantation was unnecessary. Many said they might reconsider if dialysis access problems arose or their health declined, but at the time of the interview, they were content.

Philip, Tomos, Adam, Owain, Peter and Hannah described dialysis as just part of their routine:

You get up, you go to work [dialysis unit], you help the machine work. You come home, job done. Forget about it for the rest of the day. (Owain)

Observing long‐term dialysis patients reassured younger people that they could also live long lives on dialysis. Owain had been on dialysis for nearly 30 years—longer than some nurses had worked on his unit. Tomos took comfort from seeing others on dialysis for decades:

There's a guy at my unit—he's been on dialysis for 37 years! That would take me to nearly 80.

Dialysis units were described as supportive communities, where patients formed close bonds with staff and each other. For some, dialysis offered more emotional support than they expected from a transplant clinic.

#### 5.9.3 Home Dialysis Having Similar Outcomes to Transplantation

Gloria, Jean, Jacqui, Richard, Gareth, Mark, Sean, Gerraint, Helen and Harry on home dialysis valued the freedom and control it offered, similar to transplant patients but without the risks of immunosuppression. Mark, on nocturnal home haemodialysis, believed his health outcomes were comparable to a kidney transplant:

I could easily do 40–50 h of treatment a week—it wouldn't impact my lifestyle at all. Why would I risk invasive surgery [kidney transplant] and immune suppression for very little gain?

## 6 Discussion

This study is the first large‐scale and rigorously conducted qualitative exploration of why people in the UK decline kidney transplantation despite being medically suitable. It provides the most comprehensive account to date of the deeply personal, complex and rational reasons behind these decisions, drawn from a large and diverse sample using and testing a theoretically informed framework. Findings highlighted a gap between policy aspirations to increase transplant uptake, clinical expectations that patients would want a transplant, and the deeply personal values and preferences of individuals making these decisions. By identifying the factors that shape transplant decision‐making throughout an individual's kidney journey, this study addresses a critical gap in the literature, offering new insights from multiple patient perspectives and testing the theory developed from the QES to contextualise and conceptualise kidney transplant decision‐making.

Building on the findings of the initial qualitative evidence synthesis (Jones et al. 2022), findings from this study found that decisions to decline transplantation were primarily driven by past experiences, personal beliefs and perceived control over treatment choices. Negative past experiences, especially trauma and previous transplant failure, reinforced fears of transplantation, and for some people, dialysis was safer and more predictable.

Novel findings also highlight for the first time the impact of external factors, particularly the COVID‐19 pandemic, on transplant decision‐making. Findings provide new insights into why some people removed themselves from transplant waiting lists due to concerns over service disruption and the uncertainty of COVID‐19 outcomes. These findings align with previous COVID‐19 research (Mackintosh et al. 2023; Manzia et al. 2021), which reported how disrupted healthcare provision generally exacerbated patient anxieties. Future research should explore how external crises influence long‐term decision‐making and what support mechanisms could mitigate their impact.

Emotional concerns, including fears of transplant failure, high treatment burden, and potential side effects of immunosuppressant medication, mirrored those reported by Ouellette et al. (2009) and Loban et al. (2023) who highlighted the psychological and emotional toll of transplant failure and the need for individuals to regain control. These findings align with Holley et al. (1996) where previous transplant recipients expressed more negative perceptions of transplantation than those who had never been transplanted.

People also described an evolving sense of adaptation and acceptance of dialysis, echoing the work of Mehta et al. (2024), in which dialysis was viewed as a stable and manageable long‐term option. Many expressed a ‘better the devil you know’ mentality, preferring dialysis over the uncertainty of transplantation. Individuals on home haemodialysis reported a sense of freedom and improved quality of life, aligning with Cases et al. (2011), who identified home dialysis as a means of maintaining personal agency and lifestyle continuity.

Beyond kidney transplantation, this study draws parallels with decision‐making in other organ transplants, such as lung transplantation (Chen et al. 2023). Similarities in the study findings and findings by Chen et al. (2023) suggest people weighed the risks of transplantation against the relative stability of their current condition, underscoring a broader theme of uncertainty avoidance in transplant decision‐making.

These insights reinforce the need for patient‐centred approaches in clinical practice, ensuring that decision‐support tools and discussions address not only clinical risks and benefits but also understand the deeply personal values and experiences that ultimately shape individuals' choices.

### 6.1 Strengths and Limitations of the Work

This study provides new and novel theory‐informed insights into why people with kidney failure decline transplantation, challenging assumptions about transplant decision‐making and transplant being the best option for everyone. A key strength is the use of IPA to capture the deeply personal and complex reasoning behind decisions. The study also drew on the Theory of Planned Behaviour, enhancing understanding of how past experiences, attitudes and perceptions influence transplant choices. Including a diverse sample, including age, treatment modality and transplantation experiences, strengthens the generalisability of findings within the UK healthcare setting. Engagement with local Kidney Patient Associations proved invaluable to discuss findings, which further strengthened the study's credibility and reflexive practice. Involvement ensured the research addressed current patient needs and priorities; the findings were directly relevant to current UK clinical practice.

However, some limitations should be noted. The study was conducted in the UK where the health service is funded by taxation and not based on ability to pay. Some aspects of the findings may not be transferrable to all international healthcare systems such as those funded by insurance or work‐place benefits; however, the issues explored and experienced by participants are globally relevant, and the applicability and transferability of the findings will be of interest to the international kidney multidisciplinary team. The study captured decision‐making at a single point in time, whereas a longitudinal approach could have shown how decisions change over time, as some participants indicated they might reconsider transplantation in the future. While patient perspectives were central, further research should include family members and healthcare professionals to understand the broader social and clinical context. The majority of participants interviewed were White British, although the study included some people from ethnic minority backgrounds. Further research with a more diverse sample is needed to explore how spiritual beliefs, religious and cultural factors may influence and shape decisions to decline kidney transplantation.

### 6.2 Recommendations for Further Research

Further research is needed to deepen understanding of kidney transplant decision‐making, particularly in areas where patient perspectives, healthcare provider attitudes and systemic barriers intersect. Ongoing studies, such as Bailey et al.'s (2023) IN‐FAKT study, are exploring how patients, families and healthcare professionals navigate treatment options when a kidney transplant begins to fail. Building on this, research should investigate whether improved management of failing transplants increases patients' willingness to consider another transplant. Additionally, understanding why some individuals decline transplantation even after completing the work‐up process could inform the development of more patient‐centred approaches to transplant discussions.

A key gap exists in the development of validated Patient‐Reported Experience Measures (PREMs) specific to kidney transplantation. Current PREMs primarily focus on dialysis care, limiting their applicability to the transplant decision‐making process. Ongoing work by NHS Blood and Transplant (Jenkins et al. 2024) aims to create a kidney transplant‐specific PREM, but further research is required to evaluate how decision aids can incorporate not only clinical survival estimates but also patient values, preferences and lived experiences. Future studies should also assess how shared decision‐making models can be better integrated into clinical practice to balance comprehensive patient education with respect for autonomy. We describe future research priorities in Box 1.

BOX 1 **Future research priorities.**

- Longitudinal research is needed to investigate what influences people living with kidney failure to make decisions to provide critical insights into long‐term decision‐making processes.
- Incorporating family and carer perspectives could clarify the extent of their influence and the support they provide.
- Exploring healthcare professionals' perspectives to investigate how potential biases, communication barriers, or systemic factors influence patient‐provider interactions.
- Future research must prioritise equity, diversity and inclusion to ensure findings represent all populations affected by kidney disease and transplantation.
- Understanding decisions to decline repeat transplants to understand people's concerns, past experiences and expectations shaping these decisions.
- Urgent need to create and validate tools that assess patient expectations and readiness for kidney transplantation.

### 6.3 Implications for Policy and Practice

There is a need for a more patient‐centred approach to kidney transplant decision‐making, ensuring that clinical discussions and decision‐support tools address not only medical risks and benefits but also patients' values and concerns. Notably, none of the people in this study reported using formal decision‐making aids, reinforcing recent findings that current tools do not fully address the emotional and experiential factors influencing decisions (Stacey et al. 2024). While survival prediction tools like *iChoose Kidney* provide clinical data, they do not explore the deeper personal reasons behind treatment choices.

A transplant‐specific decision‐support tool is needed to inform the development of personalised care and tailored support. The enhanced theoretical framework (Figure 3) can be used by the kidney healthcare team to inform patient assessments to identify, explore, and understand individual behaviours that shape people's attitudes and beliefs towards kidney transplantation. Kidney nurses play an integral role in supporting patients and providing information about kidney replacement treatments; using the model could help develop targeted and personalised information. Over a longitudinal period, some people may change their mind, and kidney replacement decisions, including kidney transplant decisions, need to be revisited.

To bridge these gaps, policymakers and practitioners should focus on the following key areas described in Box 2.

BOX 2

- Enhanced training is needed to respect patients' autonomy in kidney transplantation decisions.
- Patients with mental capacity should receive personalised transplant information to weigh risks and benefits based on their circumstances, even if their choices contradict national policies or clinician preferences.
- Shared decision‐making tools should be used to encourage informed and collaborative discussions. Decision‐support materials must offer balanced, personalised, and accessible information to increase patient engagement.
- Continuous audit and documentation of each patient's transplant decisions, including rationales, should be implemented and shared across kidney care teams for coordinated, patient‐centred care.
- Patients who express specific fears or misunderstandings about transplantation should have access to counselling and referrals to clinical psychology for complex issues.
- Peer support from individuals with experience in dialysis and transplantation can help patients navigate their options.
- Improving kidney care standards and addressing perceptions of poor care and miscommunication are crucial in influencing transplant decision‐making. Improving the overall standards of care for individuals with kidney disease, especially regarding patient engagement and education, could help alleviate concerns that contribute to the declining transplantation.
- Better patient education and risk communication to provide clear and balanced information on the benefits and risks of transplantation can ease anxiety and misinformation.
- Personalised clinical guidelines should acknowledge the complexities of transplant decision‐making, focusing on shared decision‐making and tailoring education to individual needs.
- The application of the theoretical model may not fully capture how every individual makes a kidney replacement decision.

From a patient perspective, it is crucial clinicians recognise kidney transplantation is not the right option for everyone and is just one kidney replacement treatment available. People have a legal right to exercise their decision and decline kidney transplantation if they have the competence to understand the information provided, even if the benefits appear to outweigh their current treatment. Findings from this study revealed pressurising people to make an alternative decision contributed to poor relationships with kidney healthcare teams. When people felt pressured to consider alternative treatment's they felt their views were not heard and their values not respected. Information was biased towards kidney transplantation, how risks were communicated left people feeling afraid a transplant would leave them worse off.

It is unclear whether, even if all of the recommendations were implemented, people would change their minds. Using findings from this research, kidney healthcare teams will be able to better understand people's decision‐making. There is a need to challenge paternalistic medical models of care and support people to make decisions they feel are right for them. If every suitable individual wanted a kidney transplant, there would be an ethical dilemma, as there are currently not enough available organs to meet the demand.

## 7 Conclusion

People living with kidney failure face a series of kidney replacement decisions. Implementation of the refined theoretical framework developed from this study can potentially help identify value‐based preferences and inform shared decision‐making between patients and clinicians, leading to transformed individualised patient care. Every person reflected on and recalled past negative experiences; the theoretical framework that we developed could be utilised by clinicians to reveal and understand how individual personal affective and cognitive attitudes negatively impact and subsequently shape perceptions towards kidney transplantation. All people feared kidney transplant failure, death after kidney transplant surgery, and complications associated with kidney transplantation. People were negatively influenced by others on dialysis and in the kidney community who had failed transplants. Age‐biased perceptions of risk need to be explored and mitigated. Accurate age‐specific information which reports on individualised risks and benefits can support people to make informed decisions. Understanding what ‘best treatment’ means for individuals is needed to explore personal views on quality versus quantity of life and personal values and preferences.

## Author Contributions

The original research proposal was developed by renal clinical psychologists (K.S.) and renal consultants from Betsi Cadwaladr University Health Board in collaboration with J.N. Wales Kidney Research Lead, Bangor University. E.J. was responsible for the acquisition of data. E.J., J.N., L.M., and K.S. made substantial contributions to data analysis and interpretation of data. E.J., L.M., K.S., and J.N. were involved in drafting the manuscript and revising it critically for important intellectual content and given final approval of the version to be published. J.N., L.M., and K.S. supervised E.J. throughout the research study.

## Conflicts of Interest

The authors declare no conflicts of interest.

## Supporting information


**File S1:** Demographics taken for each of the participants.

**File S2:** Reasons for declining participation.

**File S3:** Interview schedule.

## Data Availability

The data that supports the findings of this study is available in the Supporting Information of this article.
