# Supplementary material for: Interpretative Phenomenological Study Exploring Why People With Kidney Failure Say ‘No’ to a Kidney Transplant
Source: J Adv Nurs. 2025 Oct 21;82(6):6602–21. doi: 10.1111/jan.70301 (PMC13176721; doi:10.1111/jan.70301)
Supplement: Supplementary file 1 — File S1: Demographics taken for each of the participants. [file JAN-82-6602-s003.docx]

**Supplementary File 1**

**Demographics taken for each of the participants**

| ID participant number / pseudonym |
| --- |
| Age |
| Gender |
| Ethnicity |
| Marital status |
| Interview type |
| Length of interview |
| If someone present in interview |
| Occupation |
| Religion culture |
| State benefits |
| Education Work |
| Distance from Renal |
| Distance from Tx centre |
| Health board |
| Other language |
| Kidney Disease when they found out |
| Cause renal failure |
| Known to kidney team, or crash landed |
| Modality |
| How long on current treatment |
| Previous dialysis |
| Type of previous dialysis |
| How long on previous dialysis |
| Previous Tx |
