# Supplementary material for: Interpretative Phenomenological Study Exploring Why People With Kidney Failure Say ‘No’ to a Kidney Transplant
Source: J Adv Nurs. 2025 Oct 21;82(6):6602–21. doi: 10.1111/jan.70301 (PMC13176721; doi:10.1111/jan.70301)
Supplement: Supplementary file 2 — File S2: Reasons for declining participation. [file JAN-82-6602-s002.docx]

**Supplementary File 2 Reasons for declining participation**

Each participating site was asked to record the reasons people gave for not wanting to participate in the study.

| **Reason for non-participation** | **Number of participants** |
| --- | --- |
| Not interested in taking part in the study | 25 |
| Interested but did not return consent to be contacted form | 3 |
| Did not wish to discuss views with anyone or did not want to revisit reasons for decision-making | 1 |
| Wanted to think more about kidney transplantation before making a decision | 2 |
| Reported to be 'fed up’ talking about transplant | 1 |
| Decided they may want a transplant and were now considering kidney transplantation | 9 |
| Religious reasons for not considering a transplant and did not want to take part in the study | 1 |
| Did not feel ‘ill enough’ for a kidney transplant, did not want to decide, or discuss their reasons | 2 |
