# Supplementary material for: Interpretative Phenomenological Study Exploring Why People With Kidney Failure Say ‘No’ to a Kidney Transplant
Source: J Adv Nurs. 2025 Oct 21;82(6):6602–21. doi: 10.1111/jan.70301 (PMC13176721; doi:10.1111/jan.70301)
Supplement: Supplementary file 3 — File S3: Interview schedule. [file JAN-82-6602-s001.docx]

**Supplementary File 3 Interview schedule**

**Why do people say ‘no’ to a kidney transplant?**

**My interview: What to expect**

Thank you for agreeing to take part in this interview. I am interviewing you to better understand experiences of how people with kidney failure make decisions about kidney transplantation, and to hear more about you, your views, attitudes, and experiences that led to your decision not to have a kidney transplant. Participation in this study is voluntary.

The interview is very informal and should take approximately 60 minutes depending on how much information you would like to share. With your permission, I would like to record the interview using a digital audio-recording device and I may take some notes because I don’t want to miss any of your comments. All responses will be kept confidential, and your interview responses will be anonymised and will only be shared with research team members and we will ensure that any information we include in our report does not identify you. You may decline to answer any question or stop the interview at any time and for any reason.

• Are there any questions about the interview?

• May I take notes on our discussion?

I would like you to tell me about your experiences in your own words. You can choose what you would like to talk to us about.

- Firstly can I ask you about your experiences of having kidney disease
- Can I ask you about your experiences of making your decisions about treatment for kidney disease
- Can I ask you about your experiences and decisions which led you to decide that you did not want to have a kidney transplant
- Can I ask what were the reasons for your choice?
- Can I ask what influenced your decisions?
- Can I ask about care and support you have received outside of healthcare (e.g., charities, partner, family, friends) to help you make decisions
- Can I ask if starting dialysis (if you have started dialysis) affected your decision not to have a kidney transplant?
- Is there anything in the future that may change your decision about kidney transplantation?
- I think that’s everything I have to cover. Is there anything else you’d like to tell me or any final thoughts you’d like to follow up?
